# Clinical validation of a biopsy‐based six‐gene signature prognostic for aggressive prostate cancer

**DOI:** 10.1002/bco2.474

**Published:** 2024-12-13

**Authors:** Agnieszka Krzyzanowska, Debra F. Higgins, Stephen Barron, Tony Loughman, Amanda O'Neill, Katherine M. Sheehan, Chan‐Ju Angel Wang, Bozena Fender, Leah McGuire, Joanna Fay, Anthony O'Grady, Des O'Leary, R. William Watson, Anders Bjartell, William M. Gallagher

**Affiliations:** ^1^ Department of Translational Medicine, Division of Urological Cancers, Faculty of Medicine Lund University Lund Sweden; ^2^ OncoAssure Ltd, NovaUCD Dublin Ireland; ^3^ UCD School of Medicine, UCD Conway Institute of Biomolecular and Biomedical Research University College Dublin Dublin Ireland; ^4^ Pathology, RCSI Education and Research Centre Beaumont Hospital Dublin Ireland; ^5^ RCSI Biobank, RCSI Education and Research Centre Beaumont Hospital Dublin Ireland; ^6^ UCD School of Biomolecular and Biomedical Science, UCD Conway Institute of Biomolecular and Biomedical Research University College Dublin Dublin Ireland

**Keywords:** gene expression signature, molecular risk score, patient management, prognosis, prostate cancer, risk stratification, treatment decisions

## Abstract

**Objectives:**

This study aimed to clinically validate the six‐gene prognostic molecular clinical risk score (MCRS) for the prediction of aggressive prostate cancer in diagnostic biopsy tissue.

**Methods:**

MCRS was evaluated in prostate biopsy tissue from a Swedish cohort of men with prostate cancer (UPCA, *n* = 100). The primary outcome of adverse pathology and secondary outcomes of high primary Gleason (≥G4) and high pathological T‐stage (≥T3) were assessed by likelihood ratio statistics and area under the receiver operating characteristic curves from logistic regression models; time to biochemical recurrence was assessed by likelihood ratio statistics and C‐indexes from Cox proportional hazard regression models.

**Results:**

Biopsy MCRS was significantly prognostic (*p* < 0.0001) and added significant prognostic value to clinico‐pathological features for adverse pathology, high primary Gleason and high pathological T‐stage (*p* < 0.0001). MCRS was prognostic for biochemical recurrence and added some, albeit non‐significant, prognostic value to clinical risk stratifiers, which could reflect the low number of recurrence events in the cohort.

**Conclusion:**

Biopsy‐based MCRS improves risk stratification over standard clinical and pathological information and optimises patient management after diagnosis of prostate cancer.

## 1 INTRODUCTION

Prostate cancer (PCa) is the most common non‐skin cancer in men and the second leading cause of male cancer death worldwide.^1^ While early detection is key in assuring longer term favourable outcomes,^2^, ^3^ identifying men with aggressive PCa likely to spread to other parts of the body has proven challenging. Many clinico‐pathological features have been associated with aggressive PCa and poorer outcomes and have been combined into clinical risk‐prediction tools to aid treatment decision‐making. Prostate‐specific antigen (PSA), Gleason score and T‐stage form the basis of many clinical prediction models^4^, ^5^, ^6^; while these widely categorise patients as low, intermediate or high risk for aggressive cancer, additional molecular information from a patients' tumour tissue can improve prognostic performance.^7^, ^8^, ^9^, ^10^, ^11^

We developed a six‐gene signature combined with clinico‐pathological information, the molecular clinical risk score (MCRS), which improves risk stratification over routine clinical information.^7^ MCRS comprises four prognostic genes (*FOXM1*, *MCM3*, *MTUS1* and *TTC21B*) with roles in cell proliferation, androgen receptor signalling, invasion and metastases, two reference genes (*ALAS1, PPP2CA*) and Cancer of the Prostate Risk Assessment (CAPRA).^7^, ^12^, ^13^, ^14^ The signature was developed using radical prostatectomy (RP) tissue to predict the likelihood of aggressive disease and biochemical recurrence (BCR). Men at higher risk of these outcomes would warrant more stringent follow‐up post‐surgery and consideration of adjuvant treatments (e.g. radiotherapy or androgen deprivation therapy), whereas patients at lower risk could safely avoid the adverse effects associated with these treatments.

We sought to clinically validate MCRS in a cohort of Bx samples from men diagnosed with PCa in Sweden (UPCA, *n* = 100). We present the results of the clinical validation of MCRS in prostate Bx tissue in accordance with the REMARK guidelines.^15^

## 2 MATERIALS AND METHODS

Clinical samples: Diagnostic formalin‐fixed paraffin embedded (FFPE) Bx specimens (*n* = 124) were obtained for unscreened men referred to Skane University Hospital for an eight‐core prostate biopsy, without pre‐biopsy MRI, between 2004 and 2017, based on having ≥ 3.0 ng/mL serum tPSA or ≤ 20% fPSA or a suspicious digital rectal exam. Patients gave written informed consent and were consecutively enrolled in the Urology Prostate Cancer (UPCA) cohort.^16^ These were a subset of the previously reported UPCA RP cohort for which biopsy samples were available.^7^ The study was approved by the Lund University Ethical Committee (2016/1030, 2018/937).

RNA extraction, reverse‐transcription (RT), pre‐amplification and quantitative polymerase chain reaction (qPCR): See Supplementary Methods for details. Given the reduced tumour tissue available in a Bx core compared to an RP sample, we analytically validated a three‐step real‐time (RT)‐qPCR protocol, which incorporated a pre‐amplification reaction, to allow analysis of the six‐gene signature using a lower RNA input. Twenty‐four samples did not meet quality control after RT‐qPCR resulting in a cohort of 100 Bx samples.

Generation of molecular risk score (MRS) and MCRS: Gene expression levels were normalised and used to calculate the MRS and MCRS as described previously.^7^ Both MRS and MCRS are continuous numeric values between 0 and 10.

Statistical analysis: MRS and MCRS were validated using a predefined Statistical Analysis Plan implemented independently by two statisticians in two statistical software systems (R version 4.1.1 and SPSS version 27). The primary binary outcome was adverse pathology (AP, defined as one or more of pathological Gleason score ≥4 + 3, pathological stage ≥T3, node positivity, tertiary Gleason 5, cribriform or BCR at any time after RP). The secondary binary outcomes were high primary Gleason (≥G4) and pathological T‐stage (≥T3); the secondary time to event outcome was BCR within 5 years after RP.

Prognostic values of variables for the binary outcomes were assessed by likelihood ratio statistics (LRS), odds ratios and area under the receiver operating characteristic curves (AUCs) from univariable and bivariable logistic regression models. Prognostic values of variables for the time to event outcome were assessed by LRS, hazard ratios, C‐indexes and Kaplan–Meier survival curves from univariable and bivariable Cox proportional hazards regression models. A two‐sided LRS *p*‐value <0.05 was considered statistically significant. For categorical analyses, the MCRS was dichotomised at its median numeric value to show two groups of equal size.

## 3 RESULTS

### 3.1 Cohort characteristics

Clinical characteristics of the UPCA Bx cohort are listed in Table 1. Median age was 63.4 years (interquartile range [IQR] 59.2, 66.7 years), with a median PSA of 8.2 ng/mL (IQR 5.6, 13.0). European Association of Urology (EAU) risk distribution was 14% low risk, 56% intermediate risk and 30% high risk. CAPRA risk distribution was 28% low risk (0 to 2), 47% intermediate risk (3 to 5) and 26% high risk (6 to 10). Median time from biopsy to RP was 88 days (IQR 72, 114 days). Despite approximately 75% of the cohort having CAPRA low‐ to intermediate‐risk PCa at Bx, 79% were found to have features of AP at RP, 39% had primary Gleason grade 4 at RP and 47% were pT3 (Table 1). Median follow‐up time was 7.6 years (IQR 4.6, 9.7 years), and 20% experienced BCR within 5 years after RP.

**TABLE 1:** Clinico‐pathological characteristics of the UPCA biopsy validation cohort.

	Bx validation cohort UPCA, *N* = 100
Adverse pathology	
AP No	21 (21%)
AP Yes	78 (79%)
Missing	1
Primary Gleason grade at RP	
3	61 (61%)
4	39 (39%)
Pathological T‐stage	
T2	52 (53%)
T3	47 (47%)
Missing	1
Biochemical recurrence at 5 years	
BCR No	78 (80%)
BCR Yes	19 (20%)
Missing	3
Biopsy Gleason score	
6	36 (36%)
3 + 4	33 (33%)
4 + 3	17 (17%)
8	10 (10%)
9 or 10	4 (4%)
Pathological Gleason score	
6	16 (16%)
3 + 4	44 (44%)
4 + 3	28 (28%)
8	1 (1%)
9 or 10	11 (11%)
EAU risk categories	
Low risk	14 (14%)
Intermediate risk	55 (56%)
High risk	30 (30%)
Missing	1
CAPRA risk categories	
Low risk (0 to 2)	26 (28%)
Intermediate risk 3 to 5	44 (47%)
High risk 6 to 10	24 (26%)
Missing	6
PSA (ng/mL)	8.2 (5.6, 13.0)
Missing	5
Age at biopsy (years)	63.4 (59.2, 66.7)
Time from biopsy to RP (days)	88 (72, 114)
Follow‐up time (years)	7.6 (4.6, 9.7)
Missing	3
Molecular risk score (MRS)	3.4 (2.5, 4.0)
Molecular clinical risk score (MCRS)	3.8 (3.0, 4.9)
Missing	6

*Note*: Numeric characteristics are presented as median (interquartile range).

Abbreviations: CAPRA, Cancer of the Prostate Risk Assessment; EAU, European Association of Urology; PSA, prostate‐specific antigen; RP, radical prostatectomy; UPCA, Urology Prostate Cancer.

### 3.2 Independent clinical validation of MRS and MCRS for prediction of AP, high primary Gleason, high T‐stage and BCR

**FIGURE 1:**
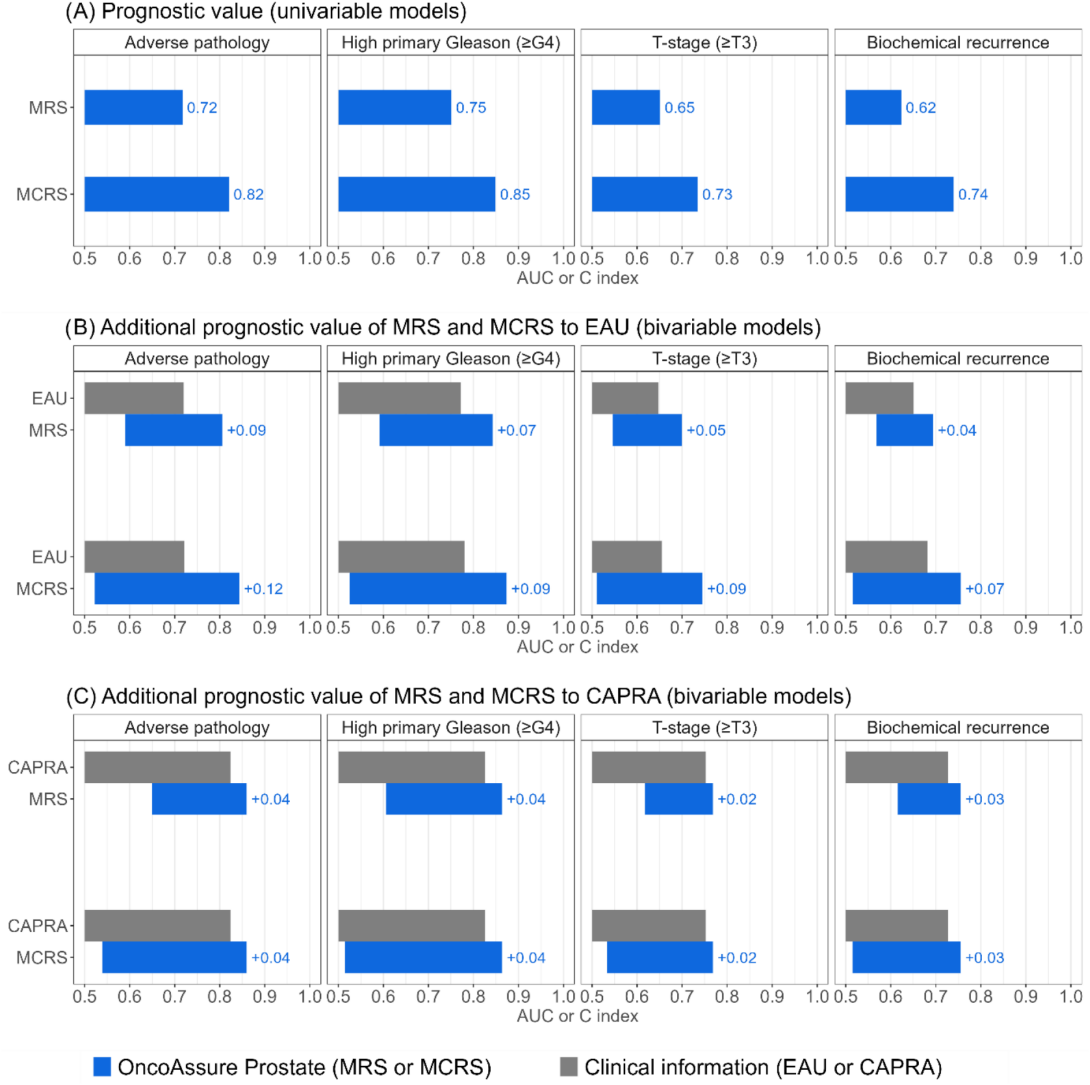
AUC values for adverse pathology, high primary Gleason, high T‐stage and C‐index for biochemical recurrence at 5 years, UPCA biopsy validation. (A) Prognostic value of MRS and MCRS for each endpoint in univariable analyses. (B) Additional prognostic value of MRS and MCRS to EAU risk categories in bivariable analyses (indicated by extension of the blue bar to the right). (C) Additional prognostic value of MRS and MCRS to CAPRA (0–10) in bivariable analyses (indicated by extension of the blue bar to the right). AUC, area under the receiver operating characteristic curve; CAPRA, Cancer of the Prostate Risk Assessment risk score (0–10); EAU, European Association of Urology risk categories (low, intermediate, and high); MCRS, molecular clinical risk score (numeric); MRS, molecular risk score (numeric); UPCA, Urology Prostate Cancer.

Molecular‐only MRS was prognostic for AP with LRS *p* = 0.0017 (Table 2) and AUC of 0.72 (Figure 1A), high primary Gleason with LRS *p* < 0.0001 (Table 2) and AUC of 0.75 (Figure 1A), high T‐stage with LRS *p* = 0.0079 (Table 2) and AUC of 0.65 (Figure 1A) and BCR with LRS *p* = 0.0451 (Table 2) and AUC of 0.62 (Figure 1A). The combined molecular and clinical MCRS showed increased AUC values for each of the endpoints, with an AUC of 0.82 for AP, 0.85 for high primary Gleason, 0.73 for high T‐stage (all LRS *p* < 0.0001) and 0.74 for BCR (LRS *p* = 0.0026).

**TABLE 2:** Likelihood ratio statistics for adverse pathology, high primary Gleason, T‐stage and biochemical recurrence, UPCA biopsy validation cohort.

	Adverse pathology		High primary Gleason (≥G4)		T‐stage (≥T3)		Biochemical recurrence	
Univariable models	LRS	LRS *p*‐value	LRS	LRS *p*‐value	LRS	LRS *p*‐value	LRS	LRS *p*‐value
MRS	9.8	0.0017	21.3	<0.0001	7.1	0.0079	4.0	0.0451
MCRS	21.8	<0.0001	38.5	<0.0001	16.9	<0.0001	9.1	0.0026

Bivariable models	Δ LRS	Δ LRS *p*‐value	Δ LRS	Δ LRS *p*‐value	Δ LRS	Δ LRS *p*‐value	Δ LRS	Δ LRS *p*‐value
MRS (independent of EAU)	5.8	0.0156	12.7	0.0004	4.0	0.0457	1.5	0.2285
MCRS (independent of EAU)	11.0	0.0009	16.2	<0.0001	8.8	0.0031	2.2	0.1345
MRS (independent of CAPRA)	4.0	0.0446	11.1	0.0009	1.7	0.1871	1.2	0.2768
MCRS (independent of CAPRA)	4.0	0.0446	11.1	0.0009	1.7	0.1871	1.2	0.2768

Abbreviations: Δ LRS, increase in likelihood ratio statistic when MRS/MCRS is added to EAU/CAPRA; CAPRA, Cancer of the Prostate Risk Assessment risk score (0 to 10); EAU, European Association of Urology risk categories (low, intermediate, high); LRS, likelihood ratio statistic; MCRS = molecular clinical risk score (numeric); MRS, molecular risk score (numeric).

The relationships between MRS and MCRS and the estimated probability of each endpoint are shown in Figure S1; men with higher MRS and/or MCRS values had higher predicted probabilities of AP (Figure S1A), high primary Gleason (Figure S1B), high T‐stage (Figure S1C) and BCR (Figure S1D).

### 3.3 MRS and MCRS improve risk stratification beyond standard clinico‐pathological features

The additional prognostic value of MRS and MCRS to routinely used clinical information was assessed using a series of four bivariable models for each of the four outcomes (16 bivariable models in total) whereby MRS or MCRS were added to either EAU risk categories or CAPRA score, and the increase in LRS (Table 2), AUC or C‐index (Figure 1B,C) assessed relative to the univariable clinical‐only model. Note that for each of the 16 comparisons, the bivariable model was based on the same set of samples as the corresponding univariable model.

For AP, MRS added statistically significant prognostic value to EAU (LRS *p* = 0.0156; Table 2) and CAPRA score (LRS *p* = 0.0446; Table 2), increasing the AUC of EAU risk categories by 0.09 (Figure 1B) and CAPRA score by 0.04 (Figure 1C). MCRS added statistically significant prognostic value to EAU risk categories (LRS *p* = 0.0009; Table 2) and CAPRA score (LRS *p* = 0.0446; Table 2), increasing the AUC by 0.12 (Figure 1B) and 0.04 (Figure 1C) respectively.

For high primary Gleason, MRS added statistically significant prognostic value to EAU (LRS *p* = 0.0004; Table 2) and CAPRA score (LRS *p* = 0.0009; Table 2), increasing the AUC of EAU risk categories by 0.07 (Figure 1B) and CAPRA score by 0.04 (Figure 1C). MCRS added statistically significant prognostic value to EAU risk categories (LRS *p* < 0.0001; Table 2) and CAPRA score (LRS *p* = 0.0009; Table 2), increasing the AUC by 0.09 (Figure 1B) and 0.04 (Figure 1C) respectively.

For high T‐stage, MRS added statistically significant prognostic value to EAU (LRS *p* = 0.0457; Table 2) but not CAPRA score (LRS *p* = 0.1871; Table 2). MRS increased the AUC of EAU risk categories by 0.05 (Figure 1B) and CAPRA score by 0.02 (Figure 1C). MCRS added statistically significant prognostic value to EAU risk categories (LRS *p* = 0.0031; Table 2) and added non‐statistically significant prognostic value to CAPRA score (LRS *p* = 0.1871; Table 2), increasing the AUC by 0.09 (Figure 1B) and by 0.02 (Figure 1C) respectively.

For BCR at 5 years, MRS added non‐statistically significant prognostic value to EAU (LRS *p* = 0.2285; Table 2) and CAPRA score (LRS *p* = 0.2768; Table 2), increasing the C‐index of EAU risk categories by 0.04 (Figure 1B) and CAPRA score by 0.03 (Figure 1C). MCRS added non‐statistically significant prognostic value to EAU risk categories (LRS *p* = 0.1345; Table 2) and CAPRA score (LRS *p* = 0.2768; Table 2), increasing the C‐index by 0.07 (Figure 1B) and by 0.03 (Figure 1C) respectively.

### 3.4 MCRS identifies patients with aggressive disease at increased risk for recurrence

To assess the clinical impact of MCRS, patient samples were categorised as lower‐risk if MCRS was below the median MCRS value or higher‐risk if it was above. All but two CAPRA low‐risk cases (22/24) were categorised as MCRS lower risk and none experienced BCR; 50% (22/44) CAPRA intermediate‐risk cases were MCRS higher risk with an increased rate of BCR (36.4% [8 of 22] vs. 13.6% [3 of 22] in MCRS lower risk cases); all but two CAPRA high‐risk cases were MCRS higher risk, and all six BCR events occurred in the CAPRA high‐risk MCRS higher risk group (Table 3).

**TABLE 3:** Reclassification of CAPRA risk groups by median‐MCRS, odds ratios for MCRS higher‐risk group for adverse pathology, high primary Gleason and T‐stage, and hazards ratio for biochemical recurrence, UPCA biopsy validation cohort.

No. of BCR at 5 years/total no. of patients		MCRS		
		Lower	Higher	Total
**CAPRA**	**Low** (0–2)	0 of 22	0 of 2	24
	**Intermediate** 3–5	3 of 22	8 of 22	44
	**High** 6–10	0 of 2	6 of 22	24
Total		46	46	92

Outcome		Odds or hazard ratio	95% CI	*p*‐value
Adverse pathology		6.3	2.1, 23.6	0.0024
High primary Gleason (≥G4)		11.0	4.1, 33.7	<0.0001
High T‐stage (≥T3)		3.4	1.5, 8.1	0.0055
Biochemical recurrence		5.4	1.6, 18.8	0.0081

*Note*: Patient samples were categorised on the basis of the median MCRS into higher‐risk and lower‐risk groups, and the odds ratio or hazards ratio for each outcome calculated. Abbreviations: 95% CI, 95% confidence intervals; Bx, biopsy; RP, radical prostatectomy; UPCA, Urology Prostate Cancer.

**FIGURE 2:**
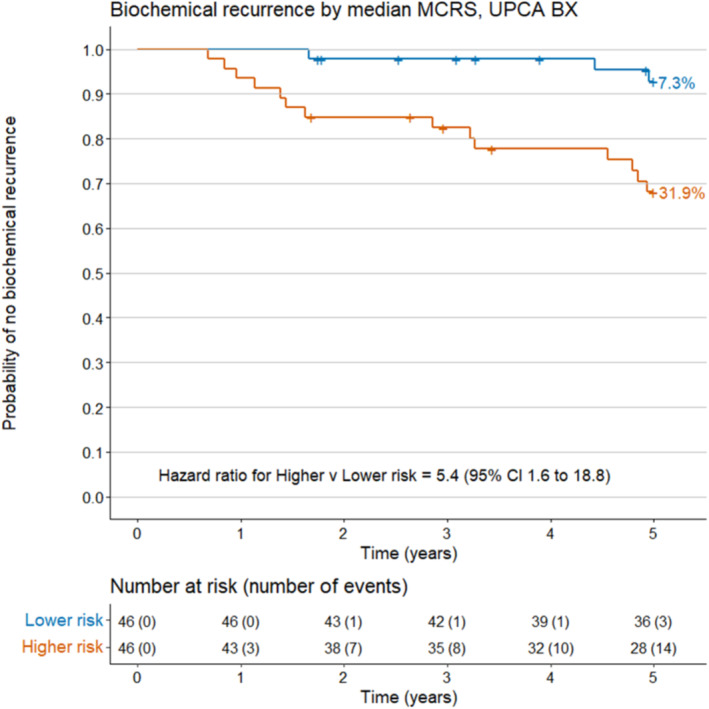
Kaplan–Meier curves for time to BCR in Bx samples (*n* = 92) categorised as lower risk (blue line) or higher risk (red line) based on the median MCRS value, UPCA biopsy validation. BCR, biochemical recurrence; MCRS, molecular clinical risk score, dichotomised at its median numeric value to show two groups of equal size; UPCA, Urology Prostate Cancer.

MCRS higher risk samples were 6.3 times more likely to have AP (odds ratio [OR] 6.3, 95% confidence interval [CI] 2.1 to 23.6), 11.0 times more likely to have high primary Gleason (OR 11.0, 95% CI 4.1 to 33.7), 3.4 times more likely to have high T‐stage (OR 3.4, 95% CI 1.5 to 8.1) and 5.4 times more likely to develop BCR (hazard ratio 5.4, 95% CI 1.6 to 18.8) (Table 3), with the probability of BCR within 5 years of RP increasing from 7.3% in patients with MCRS lower‐risk to 31.9% in those with MCRS higher risk (Figure 2). Although there were too few metastases (*n* = 5) in our cohort for reliable statistical modelling, we observed that the median value of MCRS was higher among patients with metastases than those without (MCRS = 5.0 vs 3.7 respectively; Figure S2).

## 4 DISCUSSION

We report the clinical validation of a six‐gene clinico‐pathological signature, MCRS, in prostate Bx tissue that is highly prognostic for aggressive PCa. AP is a surrogate marker of aggressive PCa and has been validated as a strong predictor of post‐treatment disease progression and 15‐year PCa‐specific mortality after RP.^17^ We report that MCRS on FFPE Bx tissue adds significant prognostic value to benchmark clinical models for AP, high primary Gleason and high T‐stage.

Current clinical risk assessment tools do not take into account the molecular information unique to each tumour.^18^ Despite ~75% of the cohort having CAPRA low‐ to intermediate‐risk PCa at Bx, 79% had features of AP at RP. This high prevalence of AP likely reflects the (i) unscreened population, (ii) absence of pre‐biopsy MRI, (iii) surgical nature of the cohort and (iv) our extensive definition of AP, which intentionally included tertiary Gleason 5, cribriform or any BCR after RP. We applied this extended definition to capture more recent features associated with aggressive PCa and poorer patient outcome. We considered that patients who developed BCR after RP were likely to harbour aggressive PCa at biopsy, which was missed by standard pathological features. With this definition, we captured an additional 17% of the cohort as having AP.

Approximately 25% of low‐risk PCa patients are upgraded on confirmatory biopsy,^19^, ^20^ highlighting the inadequacy inherent with pathological assessment of biopsy specimens. In the UPCA Bx cohort, 37% (37 of 100) were upgraded at RP, with 14% (5 of 36) of International Society of Urological Pathology grade group 1 (ISUP GG1) and 33% (11 of 33) of GG2 patients upgraded to clinically significant PCa (csPCa, GG3 to 5), while 13% (13 of 100) were downgraded. The risk of misclassification at biopsy undermines confidence in using clinico‐pathological features alone to risk stratify patients.^21^ MCRS provides additional evidence for the presence of intrinsic biological aggressiveness of a tumour without the need for further invasive testing of the patient. For example, five of 36 men with GG1 PCa at Bx were upgraded to csPCa at RP and had a mean MCRS of 4.1. In contrast, 15 of these 36 men were not upgraded at RP and had a mean MCRS of 2.5.

Genomic tests are recommended in PCa guidelines to improve risk stratification over clinical features and include the 17‐gene Oncotype Dx Genomic Prostate Score (GPS) trained on AP, the 46‐gene cell cycle progression (CCP) assay which was not trained on a specific outcome, and the 22‐gene Decipher Genomic Classifier (GC) trained on metastasis.^5^, ^8^, ^9^, ^10^ Substantial genomic heterogeneity exists in PCa^10^, ^22^ with high‐risk genomic features found in clinically low‐ and intermediate‐risk patients.^23^, ^24^ Falagario et al. reported that genomic testing further stratified men with favourable intermediate‐risk (FIR) PCa into genomic low‐ and high‐risk categories and showed that FIR men with high genomic risk had similar rates of AP as unfavourable intermediate‐risk (UIR) patients, whereas those with low genomic risk had rates similar to very low and low‐risk patients.^24^ Herlemann et al. reported that among FIR tumours, those with high Decipher risk, but not with low or intermediate Decipher risk, had increased odds for AP.^2^ In a cohort of newly diagnosed PCa patients from the Michigan Urological Surgery Improvement Collaborative (MUSIC) registry, who underwent gene expression classifier (GEC) testing, Hu et al. reported that 63% of FIR patients had GEC low‐risk disease.^25^ Similarly, we report reclassification of CAPRA risk categories by MCRS and show that MCRS higher risk patients have increased rates of BCR (Table 3). Altogether, the evidence supports the presence of men within clinical risk categories that have highly variable biological tumours, some of whom are ideal candidates for active surveillance (AS) and others who need more stringent follow‐up on AS or need to proceed with treatment sooner.^26^

The Europa Uomo Patient Reported Outcomes Study (EUPROMS) quality of life (QoL) survey, of over 3500 men with PCa across 30 countries worldwide, strongly concluded that men should consider AS as first‐line treatment to ensure the best QoL and avoid treatment‐related effects on urinary incontinence, sexual function, fatigue and insomnia.^27^ Given the highly variable rate of AS uptake,^28^, ^29^ a test which provides, at the time of diagnosis, further clarity on a patient's risk of harbouring aggressive cancer will better identify men who can safely opt for AS over immediate treatment of their PCa leading to increased AS rates. Indeed, men in the MUSIC consortium who had GEC scores below the threshold for high‐risk disease had higher rates of AS uptake (65%) versus those who had GEC scores above the threshold (16%) and versus those who did not undergo GEC testing (26%).^25^ Personalisation of active surveillance is an important aspect of patient management.^30^ A molecular test that provides reassurance to the patient on their longer‐term prognosis can help eliminate the uncertainty associated with a cancer diagnosis and optimise the timing of follow‐up visits. A reported obstacle in accessing molecular profiling information is the high cost and lack of local testing, which are partly due to the molecular methods and large panels of biomarkers analysed. MCRS, which will be commercialised as the OncoAssure Prostate Test, was designed to be performed in either local molecular pathology or reference laboratories. It will be CLIA Certified in 2024, with a plan to submit to FDA and IVDR for approval followed by decentralised distribution. The concise nature of the six‐gene assay gives advantages in manufacturing costs and complexity, which along with decentralised testing, may prove to be more economical and enable provision of accurate risk assessment to a wider patient population.

Analysing clinical samples with extensive follow‐up information (median of 7.6 years) is a major strength of our study. The relatively small sample size and the low number of BCR, metastases and death events are limitations. The low number of BCR events could reflect successful control of the disease in patients undergoing RP; that is, patients with high MRS/MCRS and aggressive PCa did not progress to BCR as surgery removed the tumour before it could spread beyond the prostate. For this reason, inclusion of more near‐term endpoints such as risk for AP, high primary Gleason and high T‐stage is an advantage. The absence of pre‐biopsy MRI in our cohort is a limitation; however, current use is aimed at pre‐biopsy risk stratification to reduce negative biopsies and increase detection of clinically significant PCa, and guidelines recommend targeted biopsy of MRI‐suspicious lesions alongside systematic biopsy to ensure adequate sampling of the prostate.^5^, ^6^

## 5 CONCLUSION

MCRS, tested on diagnostic biopsy tissue, predicts risk for aggressive disease and cancer progression better than standard clinical and pathological features, offering an additional tool for patients and their doctors to inform an optimal treatment plan.

## AUTHOR CONTRIBUTIONS

Anders Bjartell had full access to all the data in the study and takes responsibility for the integrity of the data and the accuracy of the data analysis. Study concept and design: Stephen Barron, Debra F. Higgins, Tony Loughman, Chan‐Ju Angel Wang, Des O'Leary, R. William Watson, Anders Bjartell and William M. Gallagher. Acquisition of data: Agnieszka Krzyzanowska, Stephen Barron, Debra F. Higgins, Tony Loughman, Amanda O'Neill, Katherine M. Sheehan, Chan‐Ju Angel Wang, Bozena Fender and Leah McGuire. Analysis and interpretation of data: Agnieszka Krzyzanowska, Stephen Barron, Debra F. Higgins, Tony Loughman, Amanda O'Neill, Katherine M. Sheehan, Chan‐Ju Angel Wang, Bozena Fender, Leah McGuire, Des O'Leary, R. William Watson, Anders Bjartell and William M. Gallagher. Drafting of the manuscript: Agnieszka Krzyzanowska, Stephen Barron, Debra F. Higgins, Tony Loughman, Chan‐Ju Angel Wang, Des O'Leary, R. William Watson, Anders Bjartell and William M. Gallagher. Critical revision of the manuscript for important intellectual content: Agnieszka Krzyzanowska, Stephen Barron, Debra F. Higgins, Tony Loughman, Chan‐Ju Angel Wang, Des O'Leary, R. William Watson, Anders Bjartell and William M. Gallagher. Statistical analysis: Agnieszka Krzyzanowska, Stephen Barron and Tony Loughman. Obtaining funding: Debra F. Higgins, R. William Watson, Anders Bjartell and Des O'Leary. Administrative, technical or material support: Amanda O'Neill, Joanna Fay and Anthony O'Grady. Supervision: Debra F. Higgins, Tony Loughman, Des O'Leary, R. William Watson, William M. Gallagher and Anders Bjartell. Pathology support: Katherine M. Sheehan, Joanna Fay and Anthony O'Grady.

## CONFLICT OF INTEREST STATEMENT

Anders Bjartell certifies that all conflicts of interest are the following: SB, DH, TL, CAW, BF, LMG, DOL and WMG are employees of OncoAssure. DOL and WMG are OncoAssure shareholders.

## Supporting information


**Figure S1:** Predicted probability of (A) adverse pathology, (B) high primary Gleason, (C) high T‐stage, and (D) biochemical recurrence at 5 years by MRS and MCRS, UPCA biopsy validation. The graph indicates the predicted probability (solid blue line) with 95% confidence intervals (dashed blue line). The overall prevalence of each outcome for all samples is indicated by the dotted black line. Bars show the proportion of samples in each 1‐unit bin of MRS or MCRS. MCRS = molecular clinical risk score (numeric); MRS = molecular risk score (numeric); UPCA = Urology Prostate Cancer.
**Figure S2:** Distribution of MRS and MCRS values in patients without and with metastasis, UPCA biopsy validation. Median and interquartile range for MRS and MCRS values derived from biopsy tissue are shown, dots represent MRS and MCRS values for individual cases, patients without metastases after RP (black) and patients with metastases (red) are indicated. MCRS = molecular clinical risk score (numeric); MRS = molecular risk score (numeric).

## References

[1] Centers for Disease Control and Prevention . Prostate cancer incidence by age and stage at diagnosis. Centers for Disease Control and Prevention, US Department of Health and Human Services. 2023. Available from: https://www.cdc.gov/united-states-cancer-statistics/publications/prostate-cancer.html

[2] Herlemann A , Huang HC , Alam R , Tosoian JJ , Kim HL , Klein EA , et al. Decipher identifies men with otherwise clinically favorable‐intermediate risk disease who may not be good candidates for active surveillance. Prostate Cancer Prostatic Dis. 2020;23(1):136–143. 10.1038/s41391-019-0167-9 31455846 PMC8076042

[3] Klotz L . Active surveillance for low‐risk prostate cancer. Curr Urol Rep. 2015;16(4):24. 10.1007/s11934-015-0492-z 25764118

[4] Cooperberg MR , Pasta DJ , Elkin EP , Litwin MS , Latini DM , Du Chane J , et al. The University of California, San Francisco Cancer of the Prostate Risk Assessment score: a straightforward and reliable preoperative predictor of disease recurrence after radical prostatectomy. J Urol. 2005;173(6):1938–1942. 10.1097/01.ju.0000158155.33890.e7 15879786 PMC2948569

[5] Schaeffer EM , Srinivas S , Adra N , An Y , Bitting R , Chapin B , et al. NCCN guidelines(R) insights: prostate cancer, version 3.2024. J Natl Compr Canc Netw. 2024;22(3):140–150. 10.6004/jnccn.2024.0019 38626801

[6] EAU Guidelines . Edn. presented at the EAU Annual Congress Paris 2024. ISBN 978‐94‐92671‐23‐3. Available from: https://uroweb.org/guidelines/prostate-cancer

[7] Krzyzanowska A , Barron S , Higgins DF , Loughman T , O'Neill A , Sheehan KM , et al. Development, validation, and clinical utility of a six‐gene signature to predict aggressive prostate cancer. Eur Urol Focus. 2023;9(6):983–991. 10.1016/j.euf.2023.04.004 37105783

[8] Cuzick J , Swanson GP , Fisher G , Brothman AR , Berney DM , Reid JE , et al. Prognostic value of an RNA expression signature derived from cell cycle proliferation genes in patients with prostate cancer: a retrospective study. Lancet Oncol. 2011;12(3):245–255. 10.1016/S1470-2045(10)70295-3 21310658 PMC3091030

[9] Erho N , Crisan A , Vergara IA , Mitra AP , Ghadessi M , Buerki C , et al. Discovery and validation of a prostate cancer genomic classifier that predicts early metastasis following radical prostatectomy. PLoS ONE. 2013;8(6):e66855. 10.1371/journal.pone.0066855 23826159 PMC3691249

[10] Klein EA , Cooperberg MR , Magi‐Galluzzi C , Simko JP , Falzarano SM , Maddala T , et al. A 17‐gene assay to predict prostate cancer aggressiveness in the context of Gleason grade heterogeneity, tumor multifocality, and biopsy undersampling. Eur Urol. 2014;66(3):550–560. 10.1016/j.eururo.2014.05.004 24836057

[11] Eggener SE , Rumble RB , Armstrong AJ , Morgan TM , Crispino T , Cornford P , et al. Molecular biomarkers in localized prostate cancer: ASCO guideline. J Clin Oncol. 2020;38(13):1474–1494. 10.1200/JCO.19.02768 31829902

[12] Kim MY , Jung AR , Kim GE , Yang J , Ha US , Hong SH , et al. High FOXM1 expression is a prognostic marker for poor clinical outcomes in prostate cancer. J Cancer. 2019;10(3):749–756. 10.7150/jca.28099 30719174 PMC6360432

[13] Stewart PA , Khamis ZI , Zhau HE , Duan P , Li Q , Chung LWK , et al. Upregulation of minichromosome maintenance complex component 3 during epithelial‐to‐mesenchymal transition in human prostate cancer. Oncotarget. 2017;8(24):39209–39217. 10.18632/oncotarget.16835 28424404 PMC5503607

[14] Bozgeyik I , Yumrutas O , Bozgeyik E . MTUS1, a gene encoding angiotensin‐II type 2 (AT2) receptor‐interacting proteins, in health and disease, with special emphasis on its role in carcinogenesis. Gene. 2017;626:54–63. 10.1016/j.gene.2017.05.019 28499941

[15] Altman DG , McShane LM , Sauerbrei W , Taube SE . Reporting recommendations for tumor marker prognostic studies (REMARK): explanation and elaboration. BMC Med. 2012;10(1):51. 10.1186/1741-7015-10-51 22642691 PMC3362748

[16] Braun K , Sjoberg DD , Vickers AJ , Lilja H , Bjartell AS . A four‐kallikrein panel predicts high‐grade cancer on biopsy: independent validation in a community cohort. Eur Urol. 2016;69(3):505–511. 10.1016/j.eururo.2015.04.028 25979570 PMC4643413

[17] Eggener S , Karsh LI , Richardson T , Shindel AW , Lu R , Rosenberg S , et al. A 17‐gene panel for prediction of adverse prostate cancer pathologic features: prospective clinical validation and utility. Urology. 2019;126:76–82. 10.1016/j.urology.2018.11.050 30611659

[18] Spratt DE , Zhang J , Santiago‐Jiménez M , Dess RT , Davis JW , den R , et al. Development and validation of a novel integrated clinical‐genomic risk group classification for localized prostate cancer. J Clin Oncol. 2018;36(6):581–590. 10.1200/JCO.2017.74.2940 29185869 PMC6530900

[19] Conti SL , Dall'era M , Fradet V , Cowan JE , Simko J , Carroll PR . Pathological outcomes of candidates for active surveillance of prostate cancer. J Urol. 2009;181(4):1628–1634. 10.1016/j.juro.2008.11.107 19233388

[20] Hamdy FC , Donovan JL . Fifteen‐year outcomes of the ProtecT trial for localized prostate cancer. Reply. N Engl J Med. 2023;389(1):92. 10.1056/NEJMc2306135 37407013

[21] Haywood SC , Stephenson AJ , Klein EA . Gene expression testing as a predictor of adverse pathology after radical prostatectomy: implications for choosing patients for active surveillance. Urol Pract. 2017;4(2):140–148. 10.1016/j.urpr.2016.05.001 37592673

[22] Cooperberg MR , Simko JP , Cowan JE , Reid JE , Djalilvand A , Bhatnagar S , et al. Validation of a cell‐cycle progression gene panel to improve risk stratification in a contemporary prostatectomy cohort. J Clin Oncol. 2013;31(11):1428–1434. 10.1200/JCO.2012.46.4396 23460710

[23] Cooperberg MR , Erho N , Chan JM , Feng FY , Fishbane N , Zhao SG , et al. The diverse genomic landscape of clinically low‐risk prostate cancer. Eur Urol. 2018;74(4):444–452. 10.1016/j.eururo.2018.05.014 29853306 PMC6586429

[24] Falagario UG , Beksac AT , Martini A , Cumarasamy S , Gupta A , Prasad S , et al. Defining prostate cancer at favorable intermediate risk: the potential utility of magnetic resonance imaging and genomic tests. J Urol. 2019;202(1):102–107. 10.1097/JU.0000000000000134 30730408

[25] Hu JC , Tosoian JJ , Qi J , Kaye D , Johnson A , Linsell S , et al. Clinical utility of gene expression classifiers in men with newly diagnosed prostate cancer. JCO Precis Oncol. 2018;(2):1–15. 10.1200/PO.18.00163 PMC7440129 32832833

[26] Tosoian JJ , Mamawala M , Epstein JI , Landis P , Wolf S , Trock BJ , et al. Intermediate and longer‐term outcomes from a prospective active‐surveillance program for favorable‐risk prostate cancer. J Clin Oncol. 2015;33(30):3379–3385. 10.1200/JCO.2015.62.5764 26324359 PMC4863946

[27] Venderbos LDF , Remmers S , Deschamps A , Dowling J , Carl EG , Pereira‐Azevedo N , et al. The Europa Uomo patient reported outcome study 2.0—prostate cancer patient‐reported outcomes to support treatment decision‐making. Eur Urol Focus. 2023;9(6):1024–1036. 10.1016/j.euf.2023.05.006 37268512

[28] Agrawal V , Ma X , Hu JC , Barbieri CE , Nagar H . Active surveillance for men with intermediate risk prostate cancer. J Urol. 2021;205(1):115–121. 10.1097/JU.0000000000001241 32658588

[29] Cooperberg MR , Meeks W , Fang R , Gaylis FD , Catalona WJ , Makarov DV . Time trends and variation in the use of active surveillance for management of low‐risk prostate cancer in the US. JAMA Netw Open. 2023;6(3):e231439. 10.1001/jamanetworkopen.2023.1439 36862409 PMC9982696

[30] Tomer A , Nieboer D , Roobol MJ , Bjartell A , Steyerberg EW , Rizopoulos D . Movember Foundation's Global Action Plan Prostate Cancer Active Surveillance c. Personalised biopsy schedules based on risk of Gleason upgrading for patients with low‐risk prostate cancer on active surveillance. BJU Int. 2021;127(1):96–107. 10.1111/bju.15136 32531869 PMC7818468

