# Supplementary material for: Clinical validation of a biopsy‐based six‐gene signature prognostic for aggressive prostate cancer
Source: BJUI Compass. 2024 Dec 13;6(1):e474. doi: 10.1002/bco2.474 (PMC11771492; doi:10.1002/bco2.474)
Supplement: Supplementary file 1 — Figure S1: Predicted probability of (A) adverse pathology, (B) high primary Gleason, (C) high T‐stage, and (D) biochemical recurrence at 5 years by MRS and MCRS, UPCA biopsy validation. The graph indicates the predicted probability (solid blue line) with 95% confidence intervals (dashed blue line). The overall prevalence of each outcome for all samples is indicated by the dotted black line. Bars show the proportion of samples in each 1‐unit bin of MRS or MCRS. MCRS = molecular clinical risk score (numeric); MRS = molecular risk score (numeric); UPCA = Urology Prostate Cancer. Figure S2: Distribution of MRS and MCRS values in patients without and with metastasis, UPCA biopsy validation. Median and interquartile range for MRS and MCRS values derived from biopsy tissue are shown, dots represent MRS and MCRS values for individual cases, patients without metastases after RP (black) and patients with metastases (red) are indicated. MCRS = molecular clinical risk score (numeric); MRS = molecular risk score (numeric). [file BCO2-6-e474-s001.docx]

**Supplementary Material**

**Supplementary Methods:**

*RNA extraction*: Six 5μm sections were prepared from the core containing the longest linear tumour length with the highest Gleason grade. Section 1 was H&E stained and reviewed by an expert uropathologist (KS). Tumour regions were annotated, assigned a Gleason grade and presence of cribriform pattern noted. Unstained Bx sections were macro-dissected to enrich for prostate adenocarcinoma tissue and RNA was extracted as previously described (7).

*Reverse transcription (RT)*: RNA (25 - 75 ng) was converted to complementary DNA (cDNA) using the High-Capacity cDNA Reverse Transcription Kit with RNase inhibitor (Applied Biosystems). Reactions were performed in a final volume of 20 μL and contained specific reverse primers for each of the six genes at 100 nM per oligonucleotide. Oligonucleotides were purchased from Integrated DNA Technologies. The RT reaction was incubated at 25°C for 10 min, 37°C for 120 min and 85°C for 5 min using a SimpliAmp thermal cycler (Applied Biosystems).

*Pre-amplification*: cDNA was pre-amplified using TaqMan PreAmp Master Mix (Applied Biosystems). Reactions were performed in a final volume of 30 μL and contained specific forward and reverse primers for each of the six genes at 37.5 nM per oligonucleotide. Oligonucleotides were purchased from Integrated DNA Technologies. The pre-amplification reaction was incubated at 95°C for 10 min followed by 10 cycles of 95°C for 15 sec and 60°C for 4 min, and a final inactivation step of 10 min at 99°C, using a SimpliAmp thermal cycler (Applied Biosystems).

*qPCR*: Hydrolysis probe gene expression assays (Integrated DNA Technologies) were manufactured with a primer-to-probe ratio of 3:1 and spotted in MicroAmp Fast Optical 96-well reaction plates (Applied Biosystems). PCR reaction mixes were prepared by combining pre-amplified cDNA samples and TaqMan® Fast Advanced Master Mix (Applied Biosystems). PCR reactions were performed in a final volume of 20 μL per assay well with 0.19 μL pre-amplified cDNA per reaction. Cycling parameters were 50°C for 2 min, 95°C for 2 min followed by 40 cycles of 95°C for 3 sec and 60°C for 30 sec. Data acquisition was performed during the annealing and extension step at 60°C. Pre-amplified cDNA samples were tested in triplicate per gene expression assay in each run. A positive control human total reference RNA (Agilent Technologies) and a negative no template control were subjected to RT, pre-amplification and qPCR steps in parallel for each batch of test samples processed.

**
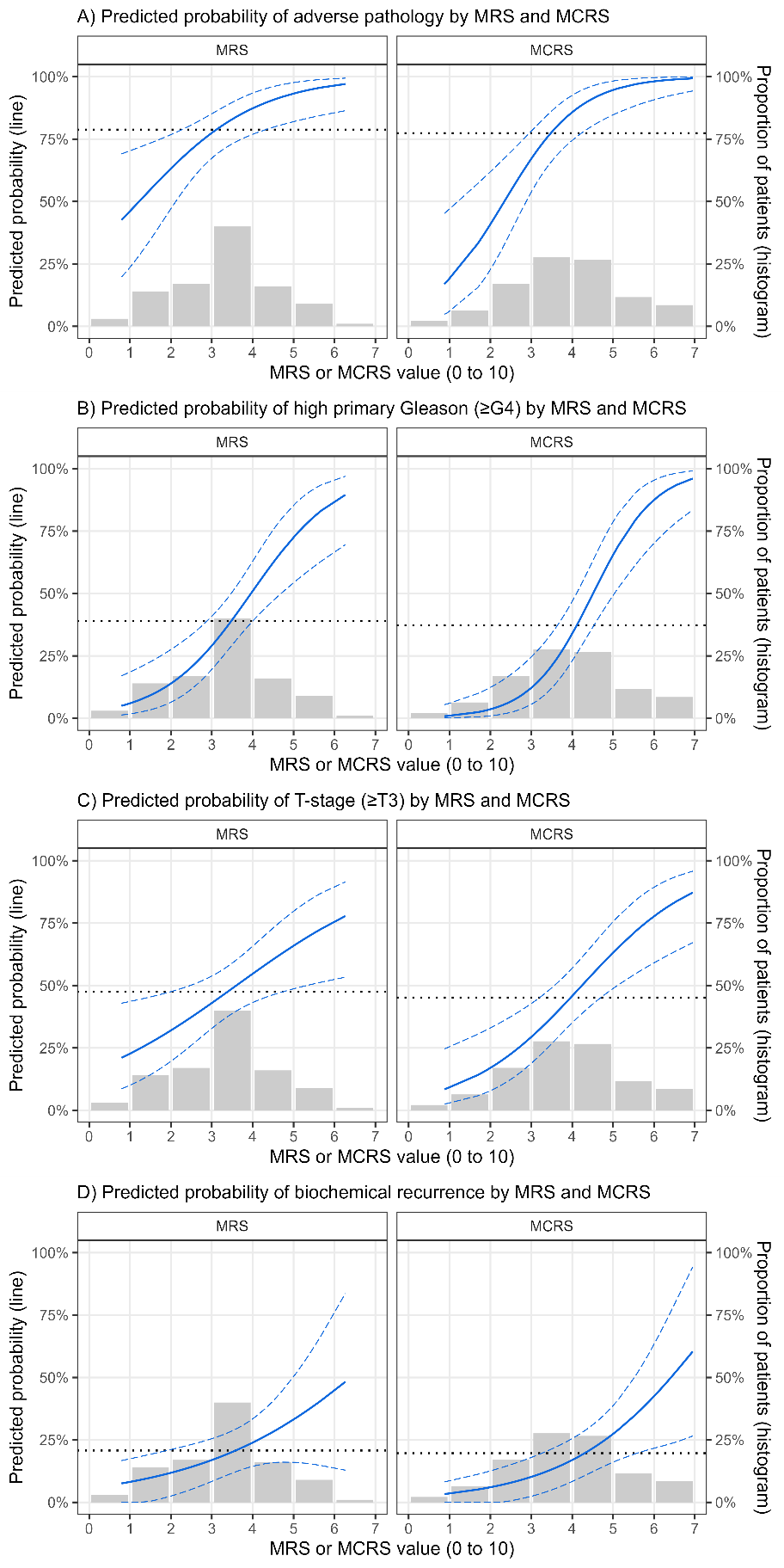
**

Supplementary Figure 1: Predicted probability of (A) adverse pathology, (B) high primary Gleason, (C) high T-stage, and (D) biochemical recurrence at 5 years by MRS and MCRS, UPCA biopsy validation. The graph indicates the predicted probability (solid blue line) with 95% confidence intervals (dashed blue line). The overall prevalence of each outcome for all samples is indicated by the dotted black line. Bars show the proportion of samples in each 1-unit bin of MRS or MCRS. MCRS = molecular clinical risk score (numeric); MRS = molecular risk score (numeric); UPCA = Urology Prostate Cancer.


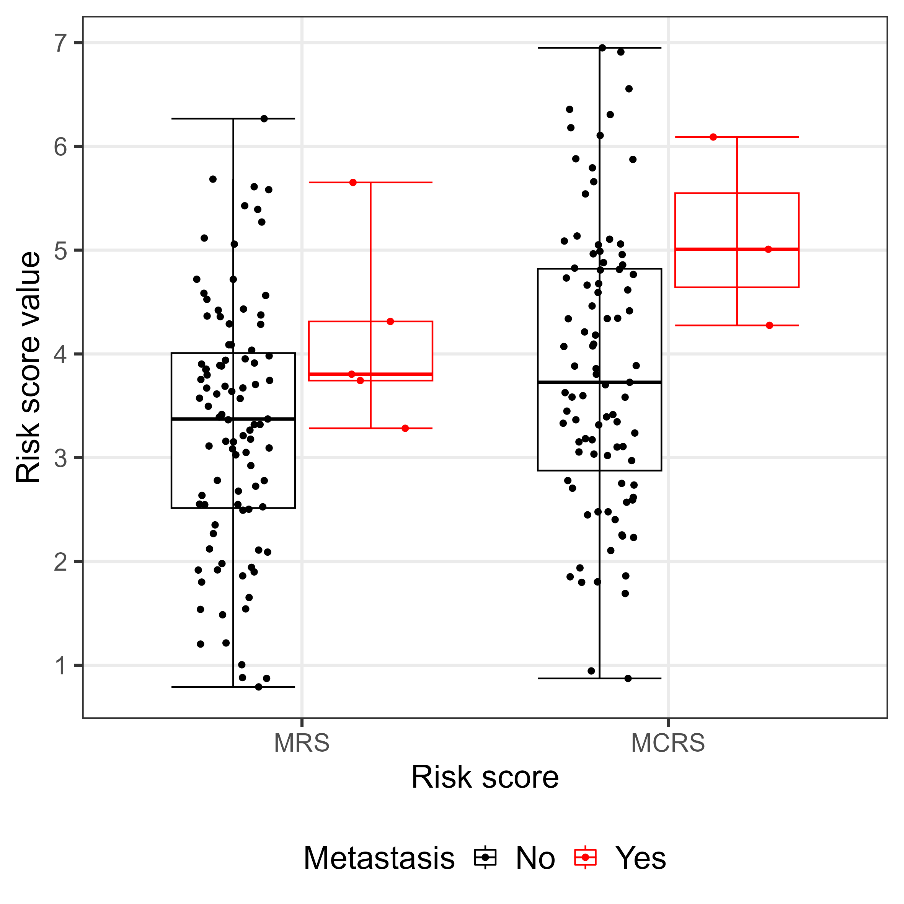


Supplementary Figure 2: Distribution of MRS and MCRS values in patients without and with metastasis, UPCA biopsy validation. Median and interquartile range for MRS and MCRS values derived from biopsy tissue are shown, dots represent MRS and MCRS values for individual cases, patients without metastases after RP (black) and patients with metastases (red) are indicated. MCRS = molecular clinical risk score (numeric); MRS = molecular risk score (numeric).
